# Collective electrical oscillations of a diatom population induced by dark stress

**DOI:** 10.1038/s41598-018-23928-9

**Published:** 2018-04-03

**Authors:** Paulo R. F. Rocha, Alexandra D. Silva, Lia Godinho, Willem Dane, Pedro Estrela, Lode K. J. Vandamme, Jose B. Pereira-Leal, Dago M. de Leeuw, Ricardo B. Leite

**Affiliations:** 1 Department of Electronic and Electrical Engineering, University of Bath, Claverton Down, Bath, BA2 7AY United Kingdom; 2 IPMA – Instituto Português do Mar e da Atmosfera, I. P., Av. Brasília, 1449-006 Lisboa, Portugal; 3 CCMAR – Centre for Marine Sciences, Universidade do Algarve, Campus de Gambelas, Ed.7, 8005-139 Portugal; 4 Electrical Engineering, Eindhoven University of Technology, P.O. Box 513, 5600 MB Eindhoven, The Netherlands; 5 Instituto Gulbenkian de Ciência, Rua da Quinta Grande n°6, 2780-343 Oeiras, Portugal; 6 Faculty of Aerospace Engineering, Delft University of Technology, Kluyverweg 1, 2629 HS Delft, The Netherlands

## Abstract

Diatoms are photosynthetic microalgae, a group with a major environmental role on the planet due to the biogeochemical cycling of silica and global fixation of carbon. However, they can evolve into harmful blooms through a resourceful communication mechanism, not yet fully understood. Here, we demonstrate that a population of diatoms under darkness show quasi-periodic electrical oscillations, or intercellular waves. The origin is paracrine signaling, which is a feedback, or survival, mechanism that counteracts changes in the physicochemical environment. The intracellular messenger is related to Ca^2+^ ions since spatiotemporal changes in their concentration match the characteristics of the intercellular waves. Our conclusion is supported by using a Ca^2+^ channel inhibitor. The transport of Ca^2+^ ions through the membrane to the extracellular medium is blocked and the intercellular waves disappear. The translation of microalgae cooperative signaling paves the way for early detection and prevention of harmful blooms and an extensive range of stress-induced alterations in the aquatic ecosystem.

## Introduction

Diatoms are a highly diverse and abundant group of phytoplankton in the aquatic environment. As photosynthetic organisms, diatoms are responsible for approximately 40% of the net marine primary production, form the basis of many marine food webs and play a key ecological role in the bio-geochemical cycling of carbon and silica. Their evolutionary and ecological success in contemporary oceans suggests that diatoms can monitor the environment and adapt to its physicochemical changes. For instance, diatoms can sense motility^1^, O_2_^2^_,_ light^3,4^ and temperature shocks^5–7^. Evidence that diatoms adapt to sudden changes in the environment are chemoperception, cell defense^3,8–10^ and, especially, the ability to collectively bloom and to adjust their sinking kinetics to enhance photosynthesis.

Many of these processes are collective phenomena where cell-cell signaling is a cooperative effect; meaning that the diatoms release a chemical signal that is perceived and amplified by others, hereby spreading a message throughout the whole population. A number of key studies suggest a dominant role of Ca^2+^ ions in downstream diatom cell-cell paracrine signaling. For instance, aequorin-transformed *Phaeodactylum tricornutum* diatoms show a rapid increase in concentration of cytosolic Ca^2+^ ions in response to mechanical stimuli and to nutrients such as Fe^11^. In algae the chloroplast Ca^2+^ flux depends on different light-dark exposures^4,11^.

Although it is assumed that Ca^2+^ ions are important intracellular messengers in diatoms, little is known about their specific role at the cellular level. As a versatile technique to investigate diatoms and their communication we measured their electrical activity. Electrophysiology in marine algae cells has surprisingly received little attention, even though the first study goes back to the early 20^th^ century^12^. Only a few subsequent studies have probed ion fluxes in single diatoms^13–17^. However, a population of diatoms has not yet been investigated.

The electrical detection of cells is commonly recorded using multi electrode arrays (MEAs). They comprise multiple planar electrodes on a substrate in close contact with cells in a culture medium; arrays with 10^4^ electrodes with area of 30 µm^2^, each only tens of microns apart have been reported^18,19^. MEAs record the extracellular field potential, which consists of a superposition of voltage gated, or ligand gated ion channels, and intrinsic membrane fluctuations. Collective phenomena show up when the signals of the numerous discrete electrodes appear synchronized in time. However, conventional MEA systems are designed to record fast varying action potentials in neurons, and therefore use operational bandwidths of at least 1 kHz. Consequently, low frequency biological oscillations are filtered out; their detection is impaired or even inhibited. To circumvent this constraint, in this work, we use large area electrodes. Their low impedance allows low frequency measurements with improved signal-to-noise ratio. We measure the electrical response of the whole population of cells adhered to the electrode. The measured signal is then the sum of all individual cell contributions. When the activity of the cells is not coordinated, the overall signal of the whole population appears as uncorrelated noise. However, when the cells operate cooperatively, the signal appears as synchronized electrical spikes.

In this study we used *Pseudo-nitzschia fraudulenta* diatoms because of their ecological importance as a harmful algae bloom forming species. Their electrical response in light is distinctively different from that in dark. In light the diatoms are electrically silent but in complete darkness a population of diatoms exhibit pronounced quasi-periodic oscillations or intercellular waves. Here, we show that the origin of this behavior is paracrine signaling between the diatoms induced by stress. From fluorescence microscopy and by using specific inhibitors, we relate the signaling with a flow of Ca^2+^ from the chloroplast stroma to the cytosol and back to the extracellular medium.

## Experimental

### Transducer

The transducer consists of a glass substrate on which circular electrodes of 100 nm Au on top of a 4 nm Cr adhesion layer were evaporated through a shadow mask. The surface area was 6 mm^2^ and the roughness as measured with a DEKTAK surface profilometer amounted to less than 1 nm. Then a drilled PMMA well was glued on top of the substrate with the prefabricated electrodes. This well serves as a container for diatom suspension. An aliquot of 1 ml of a diatom cell suspension in standard Guillard’s (F/2) Marine Water Enrichment medium with a concentration of 1.9 × 10^5^ cells/ml was transferred in to the well. The cells were allowed to settle on to the electrodes for 2 h before any measurements were performed. The culture medium was not refreshed.

The electrodes were located inside the well and connected with a small strip-line to the contact pad outside the well. Current noise measurements were performed by a Stanford low-noise current amplifier (SRS 570). All electric noise measurements were unbiased. We used the highest possible amplification with the bandwidth as low as 20 Hz. The time resolution was then sufficient to resolve slow oscillations in the extracellular field potentials. The ultrasensitive detection system was realized using a current amplification of 5 nA/V. External interference was minimized through the use of a Faraday cage and low noise cables.

The equivalent electrical circuit and detection mechanism has been reported previously^20–22^. Low frequency electrical signals can be measured either in current or voltage, with comparable signal-to-noise ratio^20^. Here we report the activity in current for historical reasons.

### *Pseudo-nitzschia fraudulenta* culture and cell counts in growth curves

Experiments were carried out using a clonal non-axenic strain of *Pseudo-nitzschia fraudulenta*. The strain was isolated from a bloom that occurred in Cascais, Portugal, in October 2014. The inoculum was maintained in sterile-filtered seawater with F/2 medium^23^. A batch culture was initiated for biomass growth in 1 l borosilicate Erlenmeyer flask at 19 °C, 33–34 PSU, illuminated with cool-white fluorescent lights with a flux of 120 μmol.m^−2^.s^−1^ on a light-dark cycle of 12 hours each. The culture was gently homogenized before 3 ml samples were taken from each flask in triplicate, daily, for cell count. Diatom cells were maintained in controlled conditions in a FitoClima Bio 600 plant growth chamber (Aralab). Cells were preserved with Lugol’s iodine solution and counted using an inverted microscope (Leica DMi8) at 100x magnification. Only living cells were quantified with a Palmer-Maloney counting chamber^24^. The biomass was used to estimate growth phases and determine with precision the periods when cells were most viable.

### Proliferation assay

Assays were carried out in 96-well plates using the CellTiter 96 AQueous One Solution Cell Proliferation Assay according to the manufacturer’s instructions. Cells were plated in quadruplicate wells containing 100 ml of medium and GdCl_3_. The concentrations used in the viability experiments varied from 10 to 1000 µM for GdCl_3_ and cell proliferation was evaluated after 24 hours.

### Fluorescence tracking of intracellular calcium localization

*Pseudo-nitzschia fraudulenta* cells were harvested, placed in a µ-dish (Ibidi) and stained for 60 minutes with Oregon Green BAPTA-1 AM (Thermo Fisher) at 10 µM with a final concentration of 0.1% DMSO and observed immediately using a DeltaVision System. Autofluorescence was acquired in the red channel under blue excitation and Oregon Green was acquired in the GFP channel.

### Bright field and fluorescent microscopy

Image acquisition was performed using a Nikon Eclipse TE2000-S equipped with a Hamamatsu Flash 2.8 sCMOS 2.8Mpx and micromanager software for controlling the microscope and camera. ImageJ was then used for post-processing operations such as, measuring and counting cells.

### *Pseudo-nitzschia fraudulenta* diatoms

**Figure 1:**
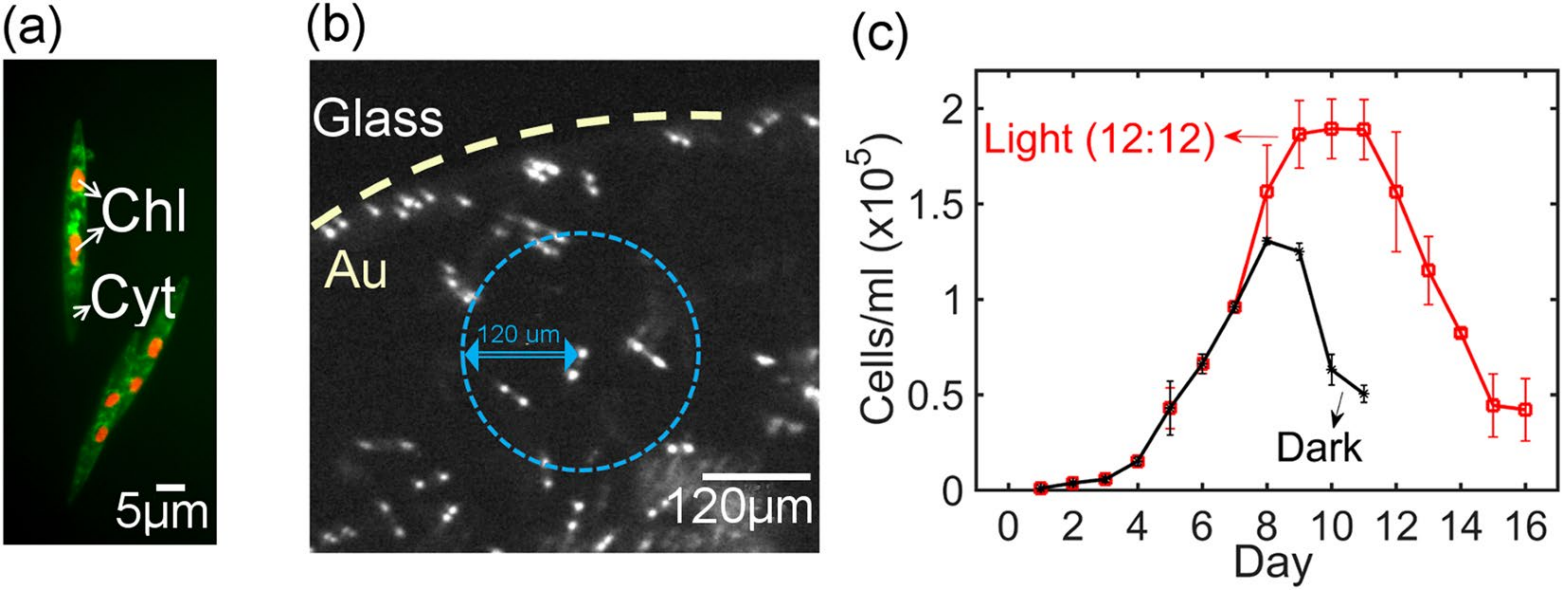
*Pseudo-nitzschia fraudulenta* diatoms. (**a**) Fluorescence image of diatoms. The orange spots indicate the choroplasts (Chl). Calcium in the cytosol (Cyt) is shown in green. (**b**) Bright field optical micrograph taken 24 hours after depositing cells on the transducer. The dotted line separates the Au electrode and the glass substrate. The blue circle is centered at a single diatom and represents an area with a radius of 120 µm. (**c**) Growth curves in triplicate. The red trace corresponds to diatoms grown at 19 °C in light-dark cycles of 12 hours each using a photosynthetic photon flux density of 120 µmol m^−2^ s^−1^. The black curve corresponds to diatoms grown first kept in light until day 4, followed by complete darkness to day 11.

*Pseudo-nitzschia fraudulenta* is a bilaterally symmetrical pennate and photosynthetic diatom, with two well distinct chloroplasts. Cell walls are made up of elongated porous frustules and comprise two overlapping sections joined by girdle bands of silica. Each cell has an average length size of about 70 µm and width of about 5 µm. The fluorescence image of two diatoms, presented in Fig. 1a, shows the chloroplasts in orange and the free calcium in the cytosol in green. The diatoms were allowed to settle on the transducer. The optical micrographs of Fig. 1b are taken 24 hours after depositing and show that the diatoms are uniformly spread across the Au electrode and glass substrate. Diatoms are separated apart by an average distance of about 120 µm. The average amount of cells settled on the sensing electrode of 6 mm^2^ is about 1000 ± 300.

Growth curves vary considerably with species, within strains and with culturing conditions such as light flux^25^. We obtained growth curves of *Pseudo-nitzschia fraudulenta* diatoms starting form an initial concentration of 1000 cells/ml. The cell medium, F/2, was not refreshed. Cells were grown at 19 °C in light-dark cycles of 12 hours each, using photosynthetically active radiation, or grow light, with a flux density of 120 µmol m^−2^ s^−1^. The growth curve, presented as the red line in Fig. 1c, shows a time lag phase where no significant increase occurs, followed by a phase where the concentration increases with time. Then there is a short period where the concentration remains constant and a decay phase where the cell concentration decreases with time. The black line in Fig. 1c represents cells grown first in light until day 4, followed by complete darkness to day 11.

The growth curve obtained using light-dark cycles of 12 hours each was similar to that reported in the literature^25^. The concentration is maximized after 7 days and amounted to 1.9 × 10^5^ cells/ml. These cells are viable for testing and applied to the transducer.

In complete darkness, cells can only grow by consuming energy stored during a previous light exposure. However, the stored energy is not refreshed and therefore the death rate increases and the growth curve is shorter as compared to cells exposed to light-dark cycles of 12 hours each. Still, the diatoms are able to survive and even grow in dark conditions. This suggests that diatoms retain their photosynthetic capacity even in dark conditions. The retention of pigments during long periods of darkness are additional evidence that diatoms retain their photosynthetic capacity in dark^26^. The photosynthesis capacity in dark suggests that photosystem II cycles by using energy from respiration^11,26^.

### Extracellular electrical recordings

The diatoms, dispersed in F/2 medium, were left to settle on the transducer for about one hour before starting the electrical measurements. Optical micrographs, *c.f*. Fig. 1b, show a uniform electrode coverage with typically ~1000 cells, on average 120 µm apart. We first measured the electrical response while illuminating the diatoms with a photosynthesis-inducing photon flux of 120 µmol m^−2^ s^−1^. Only the base line current was reproduced; under continuous illumination with grow light the diatoms were electrically silent.

The lack of electrical response is at first sight counterintuitive, as the cells are highly viable and photosynthesis is ongoing. However, the electrical signal is due to a change in extracellular field potential. The lack of electrical response in light therefore means that the potential of the diatoms is not changing. This suggests that photosynthesis is self-contained within the chloroplasts of diatoms; no ions are moving in and out to the extracellular medium that leads to electrical charging of the diatoms.

**Figure 2:**
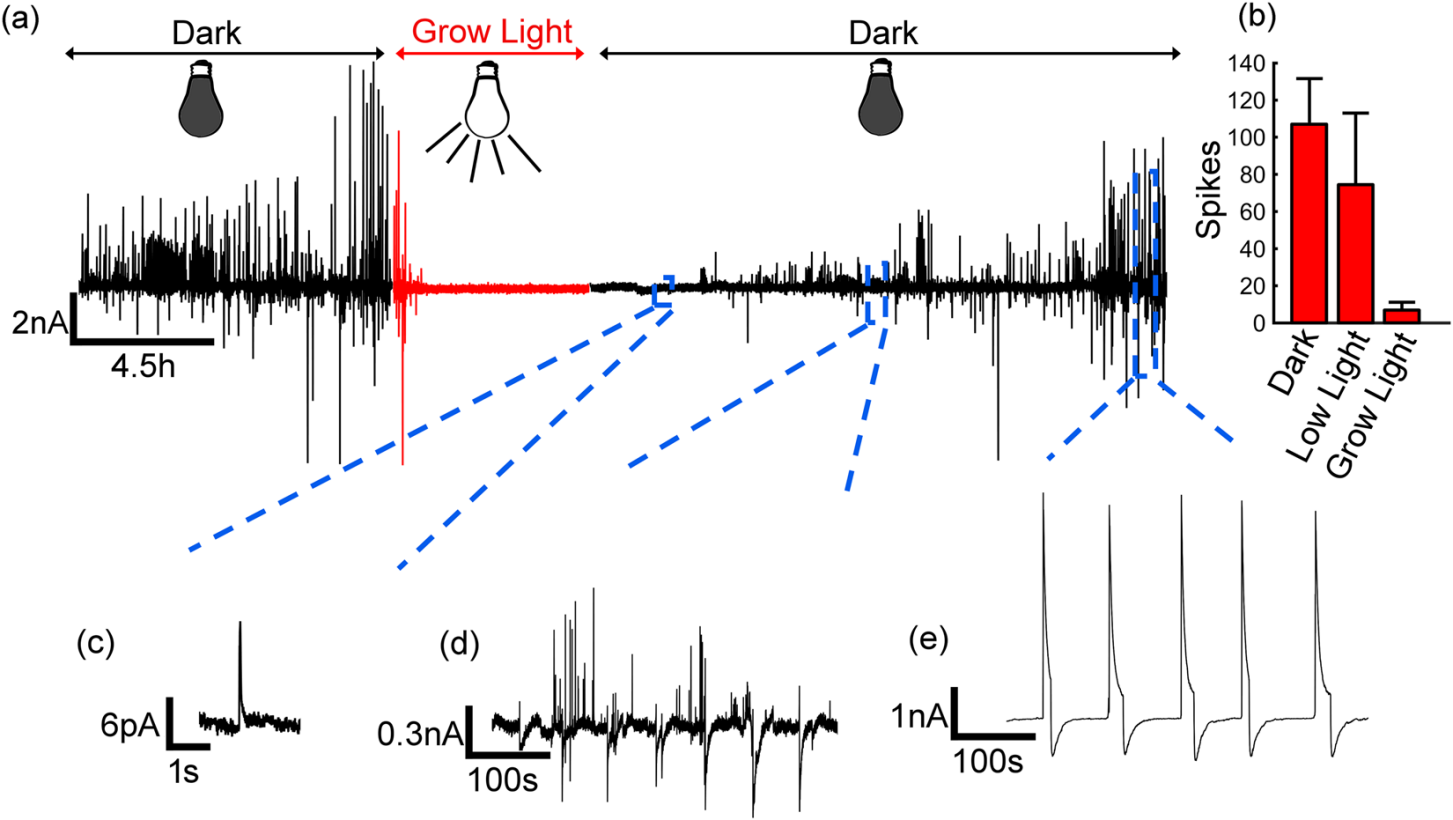
Extracellular electrical recordings of *Pseudo-nitzschia fraudulenta*. (**a**) Current response with time comprising dark and light exposure. Grow light refers to a flux of 120 µmol m^−2^ s^−1^. (**b**) Histogram of current spikes counted in 45 minutes as a function of light intensity. The current evolves from sporadic and weak, through asynchronous and bipolar signals, to strong quasi-periodic oscillations. Low light indicates a flux of 50 µmol m^−2^ s^−1^. (**c**) Magnified view of sporadic single cell spike. (**d**) Zoom in on region with asynchronous bipolar spikes (**e**) Quasi-periodic current oscillations.

We then measured the electrical activity of cells in complete darkness. First the cells were kept in light for 12 hours. Then the light was turned off and the electrical activity was measured in complete darkness. The recorded current response over a period of 16 hours is presented in Fig. 2a. With time, the signals evolve from sporadic and weak, through asynchronous and bipolar signals, to strong quasi-periodic oscillations. Figure 2b shows a magnified view of a small sporadic spike. The current is a few pA and the width about 100 ms, a typical response of a discrete diatom^13^. With time, the diatoms become more electrically active; the number of spikes and their magnitude increases, as shown in Fig. 2c. We measure all the diatoms settled onto the electrode simultaneously. The measured signal is therefore a superposition of all individual contributions. The signals of the diatoms are not synchronized, but random in time, suggesting that no communication between cells is present. The overall signal therefore shows a large spread in spike magnitude and time intervals. When the diatoms are in complete darkness for about 10 hours, quasi-periodic oscillations start to appear. A zoom in is presented in Fig. 2d. The periodicity implies that the signals of the discrete diatoms become synchronized, implying sensing and communication. From the magnitude of the current we can deduce that all diatoms are involved. There are about ~1000 diatoms on the electrode, and each diatom produces a spike up to 10 pA, *c.f*. Fig. 2b. Hence, in first approximation, the total current response when all diatoms participate is expected to be up to 10 nA, in good agreement with the actual measured current, *c.f*. Fig. 2d.

The shape of the signal resembles that of an intercellular wave^20^. In biology, there are many examples of these spatiotemporal events. The most well-known is the cardiac beating, which is due to changes in Ca^2+^ concentration that propagate through a population of myocardial cells. The speed of an intercellular wave depends on the nature and strength of the initiating stimulus as well as on the mechanism of propagation. The speed can be estimated form the pulse width. When the wave reaches the sensing electrode the conductance increases and remains high until the end of the wave. From the average pulse width of 20 s with an electrode diameter of 2.6 mm, we estimated a speed of about 130 μm/s. This value is in good agreement with reported values for intercellular waves^20,27–29^.

In short, the quasi-periodic oscillations demonstrate that the diatoms show a collective response under prolonged dark stress. The underlying mechanism is cooperative cell-cell signaling as will be discussed in the next section.

The role of light flux was investigated in light-dark cycles of 12 hours each by varying the light flux. We counted the number of electrical spikes in the first 45 min after turning on the light. When using a low light of 50 µmol m^−2^ s^−1^, the number of spikes decreased by 25% and when using grow light of 120 µmol m^−2^ s^−1^ the decrease was over 90%. The dynamic change in the electrical oscillations in diatoms, upon different light conditions, corroborates the hypothesis of a stress surveillance system among a population of diatoms^8^.

### Collective quasi-periodic current oscillations

**Figure 3:**
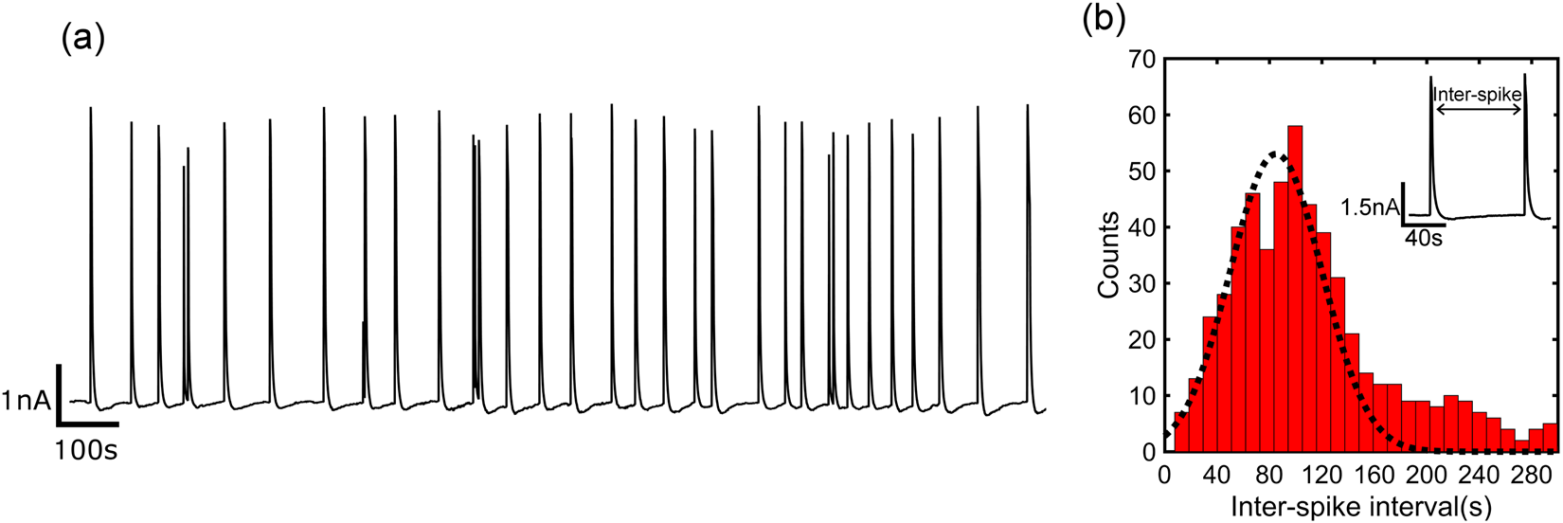
Quasi-periodic oscillations. (**a**) Extracellular electrical recording after keeping the diatoms in dark for 3 days. (**b**) Histogram of inter-spike intervals extracted from a 20 hour recording. The histogram was obtained by distributing the time intervals into 10 s wide bins. The dashed line represents a Gaussian distribution with a mean value of 80 s and a standard deviation of 30 s. The inset shows an expanded view of 2 consecutive current spikes.

The intercellular waves last for days as long as the diatoms are kept in constant darkness. A typical recording after 3 days is presented in Fig. 3a. The magnitude of the spikes is almost constant, which implies that all diatoms settled on the electrode collectively participate in each spike. The spikes are quasi-periodic. A histogram of the inter-spike distance, extracted from a 20 hours recording, is presented in Fig. 3b. The dashed line shows that the histogram can be fitted with a Gaussian distribution. The mean value of the inter-spike intervals is 80 s with a standard deviation of 30 s. This long time reflects the time scale required by the whole population to become synchronized. As diatoms are not synaptically interconnected in a network, transmission of signals probably occurs by paracrine signaling, in which one cell produces a chemical signal that diffuses to nearby cells, altering their behavior. The premises of paracrine signaling upholds recent studies on signaling mechanisms that regulate population proliferation and even programmed cell death in response to environmental changes. A few examples have been reported on the release of aldehydes such as decadienal upon zooplankton graze on diatom populations. This reduces the reproductive capacity of the grazer population by enabling an antigrazing strategy^30^. Wounding is another example where diatoms respond to treatment with the aldehyde by producing NO, a phenomenon that is modulated by intracellular Ca^2+^ gradients^21,30,31^.

**Figure 4:**
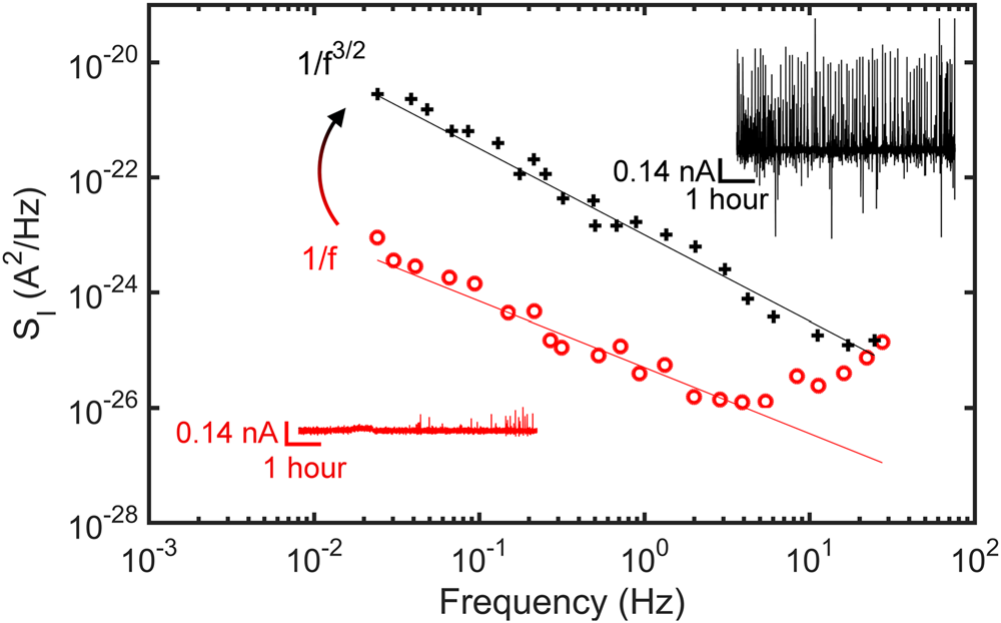
Current noise spectra of diatoms. The spectral power density, S_I_, is presented as a function of frequency, f. The red and black current traces are reproduced from the recording presented in Fig. 2. The red trace shows the random weak and sporadic signals at the beginning, and the black trace the quasi-periodic signals of the end of the recording. The noise accordingly changes from 1/f flicker noise, to diffusion limited 1/f^1.5^ noise.

We have recently shown that the spectral power of the current noise, *S*_*I*_, can reveal the underlying physics of cell-cell signaling^22,32^. Here we show that the frequency dispersion of the current noise supports paracrine signaling. As an example we took the electrical recordings presented in Fig. 2. The current as a function of time at the beginning and at the end of the recording are reproduced in Fig. 3 in red and black respectively. The beginning of the recording shows only weak and sporadic current signals while at the end of recordings, spikes dominate. For each of these recordings the current noise is calculated^33^ as *S*_*I*_*(f)* = *A*^2^*/Hz*, where A is the current magnitude at a given frequency, *f*. The current noise as a function of frequency is calculated as an average over 20 consecutive measurements and presented in Fig. 4. The functional dependence changes from 1/f to 1/f^1.5^. In the beginning the current signals are random in time, which is reflected by the characteristic 1/f flicker noise. We note that the noise increase at frequencies above 10 Hz is due to the electrochemical noise of the bare electrode^32^.

The synchronized quasi-periodic signals in the time domain lead to a 1/f^1.5^ noise in the frequency domain. This dependence is characteristic for diffusion limited noise^22,34–37^. The characteristic diffusion length, L, can be estimated from L = √Dτ where D is the diffusion coefficient and τ the inter-spike interval. As discussed in the next section we take Ca^2+^ ions as the diffusion species. Their diffusion coefficient in sea water is 7.5 × 10^−6^ cm^2^/s^38^. The majority of inter-spike intervals occurs at 80 s. Hence, with τ = 80 s, c.f. Fig. 3b, this leads to a diffusion length of 250 μm. This distance is of the same order of magnitude as the average distance between the diatoms, c.f. Fig. 1, which further suggests that the intercellular waves are due to paracrine cell-cell signaling.

The cell-cell communication is most likely due to stress. In complete darkness individual diatoms send a signal that is detected and amplified by other diatoms, until the whole population is synchronized. The signal can be a release of ions, which, concomitantly, changes the potential of the diatom. This temporary change in extracellular potential is then measured as a current spike in the electrical recording. To further test if stress is responsible for triggering a communicative process, we induced a thermal gradient. Over a period of about 3 hours we increased the temperature from 19 °C to 35 °C and back again to 19 °C. The cells remained alive and the viability after cycling was still 80% ± 10%. Importantly, the current drastically increased with temperature; the current peaks changed from 10 pA to about 240 pA and back to 10 pA. We extract from the current measurements as a function of temperature an activation energy of 1 eV, or more. This value is much larger than the activation energy for the diffusion coefficient of ions in water, which is typically around 20 kJ/Mol^39^ or 0.2 eV. The increase of current with temperature can therefore not only be due to an increase in diffusion velocity. The dominating effect presumably is an increased flux of ions with increasing temperature. Hence this experiment clearly demonstrates that cell-cell signaling is a feedback mechanism that anticipates changes in the physicochemical environment, with important implications for diatoms survival strategies.

### Role of Ca^2+^ in extracellular current oscillations

**Figure 5:**
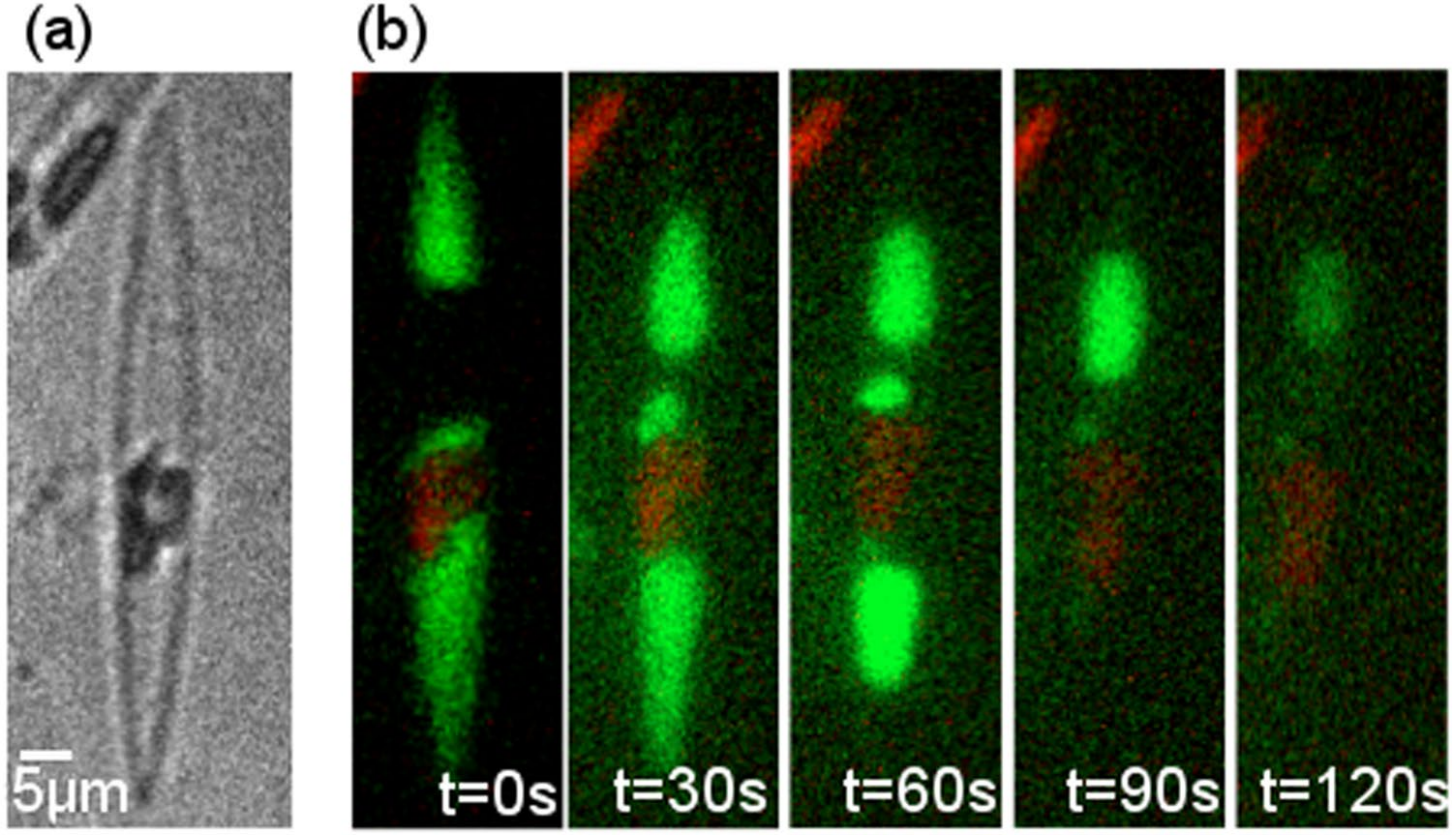
Real time Ca^2+^ diffusion pattern in a single diatom. (**a**) Bright field image showing the two chloroplast compartments enclosed by frustules. (**b**) Ca^2+^ profiles from fluorescence imaging as a function of time. In red, we see the auto-fluorescence of chlorophyll pigments. In green we see the intracellular Ca^2+^ in the cytosol vanishing with time from 0 to 120 s.

The chloroplasts organelles in diatoms can store a high concentration of Ca^2+^ ions, either bounded to the thylakoid membranes or to calcium-binding proteins^40^. The ions are stored in the chloroplasts during the light phase and released to cytosol during the dark phase. To validate our hypothesis that intercellular waves are mediated by changes in Ca^2+^ concentration we tracked the single-cell variation of Ca^2+^ concentration using fluorescence microscopy. Diatoms were stained for 60 minutes in a solution containing a selective calcium chelator. Afterwards the fluorescence intensity, which is a measure of the Ca^2+^ concentration, was imaged for a single cell. When the diatoms were kept in light, the Ca^2+^ image did not change in time. However, when the diatoms were kept in dark for 2 days before live staining, the Ca^2+^ concentration varied as a function of time and position in the diatom. A series of images is shown in Fig. 5. Figure 5a shows a bright field image of the diatom. Clearly visible are the two chloroplasts, the cytosol and the silica frustules. Figure 5b shows a series of fluorescence images as a function of time between 0 and 120 s. In the first image the Ca^2+^ ions are evenly distributed between the cytosol and the chloroplast. However, with time the overall fluorescence intensity decays meaning that the Ca^2+^ ions diffuse out of the cell. The time scale of this Ca^2+^ diffusion is comparable to the inter-spike distance of 80 s, which suggests that Ca^2+^ ions are prominently involved in the cell-cell communication and therefore mediate the electrically recorded intercellular waves. We note that an unambiguous proof of this role of Ca^2+^ ions would be the observation of a repetitive cycle of release of Ca^2+^ ions from chloroplasts to cytoplasm in and out of the cell and vice-versa. However, this observation is prevented by the photo-toxicity of the dye, which limits the viability of the diatoms over the timespan of the experiments.

**Figure 6:**
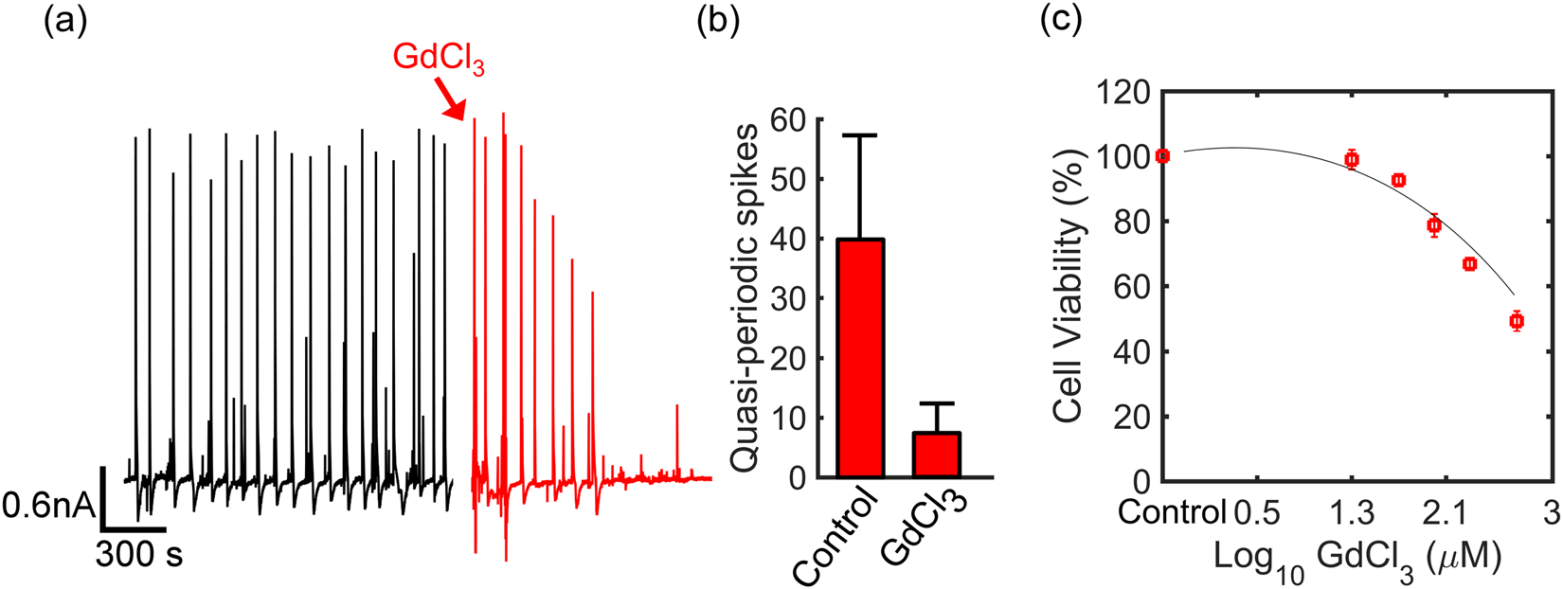
Selective blocking of Ca^2+^ stretch-activated channels. (**a**) Diatoms were kept in dark for more than 2 day/night cycles. The black trace of the current recording depicts the current fluctuations at the start of the experiment. The red trace is obtained after adding the inhibitor GdCl_3_ to the culture medium up to a concentration of 100 μM. (**b**) Data quantification recorded over 3 experiments. Current spikes were counted 45 minutes before, called control, and after adding the inhibitor, called GdCl_3_. (**c**) Cell viability in percentage upon exposure for 24 hours to different concentrations of GdCl_3_; at 100 μM there is no impact on cell viability.

The further test whether Ca^2+^ ions mediate the quasi-periodic spikes, we used the inhibitor GdCl_3_. This chemical specifically inhibits Ca^2+^ stretch-activated channels; the transport of Ca^2+^ ions through the membrane to the extracellular medium is blocked without affecting cell viability. Diatoms were kept in dark for at least 2 days. At that time, electrical recordings showed pronounced quasi-periodic oscillations, presented in Fig. 6a. Then GdCl_3_ was added to the medium up to a final concentration of 100 μM. The magnitude of the spikes immediately decreases, and after 15 minutes all oscillations are suppressed. Only the weak and sporadic asynchronous activity remains, similar to the beginning of the recording of Fig. 2a. The current spikes were counted during 45 minutes before and after adding GdCl_3_. The quantification of three experiments is presented in Fig. 6b, showing that the inhibitor completely blocked the quasi- periodic current oscillations, while not impairing cell viability (*c.f*. Fig. 6c).

## Summary and Conclusions

A population of *Pseudo-nitzschia fraudulenta* under light is electrically inert. Photosynthesis is self-contained within the chloroplasts of diatoms and does not lead to changes in the extracellular field potential. However, in complete darkness, we demonstrate that diatoms become electrically active. In a time span of hours their response evolves from weak, sporadic, uncorrelated events of single diatoms, to strong quasi-periodic oscillations synchronized by the whole diatom population. The synchronized quasi-periodic signals shows a 1/f^1.5^ frequency dependence which is normally assigned to a diffusion process. These intercellular waves last for days and are due to paracrine cell-cell signaling. Under stress, such as light deprivation, and, probably by a temperature rise, diatoms start to communicate. The cell-cell signaling is a feedback, or survival, mechanism that counteracts changes in the physicochemical environment. The messenger is likely to be related to Ca^2+^ ions as spatiotemporal changes in their concentration match the characteristics of the intercellular waves. This conclusion is supported by using the inhibitor GdCl_3_ which specifically inhibits Ca^2+^ stretch-activated channels. The transport of Ca^2+^ ions to the extracellular medium is blocked by the inhibitor and the intercellular waves disappear.

Single cell recordings such as patch clamp and fluorescent-based techniques do not allow the observation of Ca^2+^ exchange with the exterior, as Ca^2+^ quickly dissipates into the extracellular environment. However, our work on extracellular recordings of a large diatom population permits the monitoring of ions exchange between diatoms. As such, our extracellular recording approach provides a powerful indicator for the development of algae blooms and to probe ecological and physiological stress conditions in diatom populations. This can have significant implications in the prevention of harmful blooms and other alterations in the aquatic system.
